# Counterfeit *Citri Reticulatae Pericarpium* Identification: Multi‐Class Detection Using Vis/NIR Spectroscopy and Residual Neural Networks

**DOI:** 10.1002/fsn3.71975

**Published:** 2026-06-02

**Authors:** Chao Ma, Mingkun Zhang, Jianwei Ma, Sudan Chen, Yunxia Yuan, Mingtong Du, Zhiyong Dai

**Affiliations:** ^1^ College of Information Engineering Henan University of Science and Technology Luoyang China; ^2^ College of Horticulture and Plant Protection Henan University of Science and Technology Luoyang China; ^3^ College of Food and Bioengineering Henan University of Science and Technology Luoyang China; ^4^ College of Computing and Data Science Nanyang Technological University Singapore Singapore; ^5^ Complex Systems Intelligent Control Laboratory Shanghai Jiao Tong University Shanghai China

**Keywords:** Citri reticulatae pericarpium, counterfeit, food detection, machine learning, spectral

## Abstract

The expanding market demand and high commercial value of *Citri Reticulatae Pericarpium* (CRP) have led to frequent counterfeiting. Rapid and non‐destructive identification of counterfeit CRP is therefore essential. This study proposes a novel detection model that integrates visible/near‐infrared (Vis/NIR) spectroscopy with deep learning algorithms. Authentic CRP and four types of counterfeit CRP samples were collected, and their multispectral Vis/NIR data were acquired. Wasserstein Generative Adversarial Network with Gradient Penalty (WGAN‐GP) data augmentation enhanced model generalization, further improving classification robustness. After spectral preprocessing, deep learning models were applied to classify and detect different CRP types, aiming to identify the optimal network for counterfeit discrimination. The experimental results demonstrated that the Inception‐ResNet model achieved the test set accuracy of 96.56% in identifying authentic CRP from four counterfeit categories, outperforming conventional machine learning approaches. This study provides a precise and efficient model for non‐destructive authentication and adulteration analysis of CRP.

## Graphical abstract

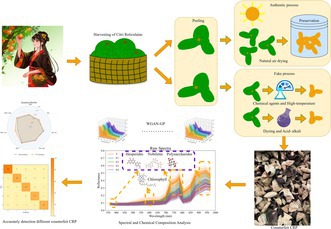

Citri Reticulatae Pericarpium (CRP) samples undergo authentic processing through natural air drying and preservation, whereas counterfeit CRP can be produced through high‐temperature treatment, chemical agents, dyeing, or acid–alkali processing. Vis/NIR spectral analysis combined with WGAN‐GP augmentation and Inception‐ResNet classification captures spectral changes related to key chemical constituents and enables accurate discrimination of different counterfeit CRP samples.

## 1 Introduction

*Citri Reticulatae Pericarpium* (CRP) has been consumed for over a thousand years as both food and medicine, valued for its distinctive aroma and flavor (Li et al. 2024; Xia et al. 2024). Beyond culinary use, CRP has demonstrated therapeutic functions, including the improvement of respiratory and digestive health (Jiang et al. 2023; Wang et al. 2024). As the dried peel of *Citrus reticulata* from the Rutaceae family, CRP must undergo a series of processing steps, including peeling, sun‐drying, and long‐term storage, before it can be marketed as an authentic product with preserved bioactivity (Sun et al. 2023; Chen, Fu, et al. 2024). This processing is intricate and requires extended periods to achieve the desired quality (Zhong et al. 2025; Yu et al. 2018).

**FIGURE 1:**
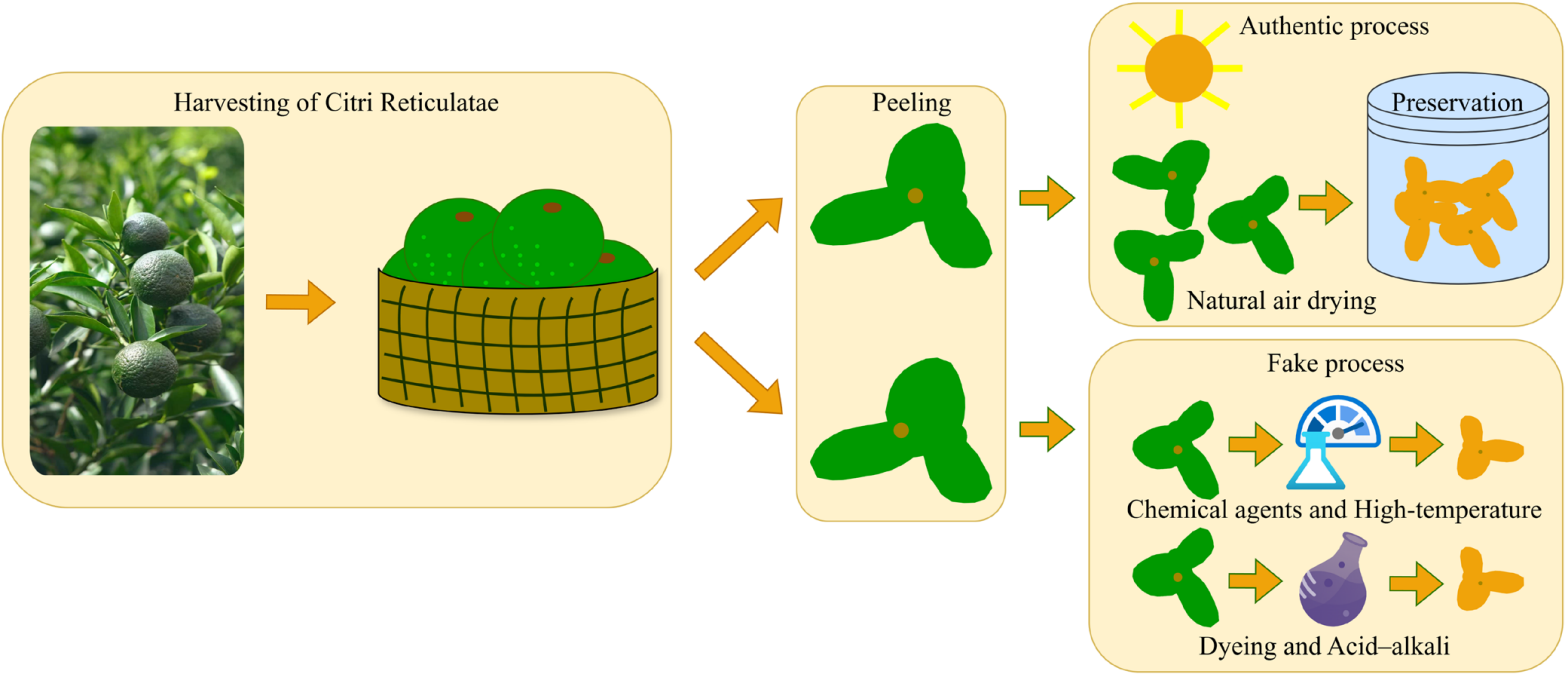
Schematic of counterfeit CRP.

The widespread use of CRP and the complexity of its processing have kept its market price consistently high. Consequently, counterfeit practices, such as process imitation and dyeing, have become increasingly common, with counterfeit CRP often presented as genuine products (Wang, Zhang, et al. 2025). These counterfeit CRPs are treated to closely resemble authentic CRP in both appearance and aroma, making them difficult to distinguish by conventional means. As illustrated in Figure 1, the schematic diagram of counterfeit CRP is presented. Such counterfeiting not only results in substantial economic losses for consumers but also poses potential health risks (Yan et al. 2025; Liu, Fu, et al. 2025). Therefore, reliable identification of counterfeit CRP has become an urgent focus of current research.

In current research, chemical analysis methods can effectively identify counterfeit CRP by quantifying constituents such as flavonoids and polysaccharides (Chen, Xiao, et al. 2024; Tao et al. 2024; Luo et al. 2018). However, these techniques are limited by their destructive nature and the inability to provide rapid detection (Li et al. 2021). Since CRP must retain its integrity and cleanliness during trade and use, the development of non‐destructive approaches for detecting counterfeits is of particular importance (Liu et al. 2023).

Visible/near‐infrared (Vis/NIR) spectroscopy can capture diverse spectral responses of a sample across multiple wavelengths (Tan et al. 2025; Pires et al. 2022; Sekhon et al. 2024). It has already been widely applied in detection and analysis across various fields (Zhang et al. 2024; Wu et al. 2025; Liu, Zhang, et al. 2025). In food research, numerous studies have employed Vis/NIR spectroscopy to investigate different products and address multiple aspects of quality assessment. Spectroscopy combined with classification models has already shown promising validation in food adulteration studies (Amirvaresi et al. 2021). Recently, research continues to explore suitable models for different products—ranging from powders (Yu et al. 2022; Cai et al. 2025) and herbs (Neto and de Carvalho Lopes 2024; Qiu et al. 2024; Qiu et al. 2024) to liquids (Li et al. 2023; Wang, Wang, et al. 2025), with consistently effective results. Zhang, Zhang, et al. (2025) proposed an adulteration identification model for *Acanthopanax senticosus* based on high‐level data fusion of portable near‐infrared spectroscopy (NIR) and portable mass spectrometry (PMS). Using fuzzy algorithms to integrate complementary spectral, their fusion model achieved a prediction accuracy of 0.96. Martins et al. (2022) applied ATR‐FTIR spectroscopy with partial least squares regression to detect wheat flour adulteration in whey protein concentrate, obtaining calibration and prediction demonstrating accurate quantification in blinded tests. Lanjewar et al. (2024) developed a portable near‐infrared spectroscopy system (900–1700 nm) combined with Savitzky–Golay (SG) preprocessing, Principal Component Analysis (PCA), and machine learning models, detecting starch adulteration in turmeric powder with high predictive performance. Using Fourier Transform Near‐Infrared Spectroscopy (FTNIR) and Laser Photoacoustic Spectroscopy (LPAS) with classifiers Partial Least Squares Regression (PLSR) and Partial Least Squares Discriminant Analysis (PLS‐DA) (Sammarco et al. 2023), achieved reliable discrimination across adulteration levels and highlighted the practicality of fast, minimal‐prep analysis for *Oregano* herb authenticity. Du et al. (2021) developed an diffuse‐reflectance near‐infrared spectroscopy with a fiber‐optic probe, coupling discriminant analysis and PLS to identify and quantify corn, rapeseed, or sunflower oil adulteration in camellia oil. Combining NIR/VIS–NIR with PCA and PLSR (Zaukuu et al. 2025), discriminated adulterated from authentic bissap materials and estimated adulterant levels, highlighting practical authentication for both calyces and prepared drinks.

In CRP research, the integration of spectroscopy with machine learning models has been applied to assess storage age and quality, provided promising results, which also provides valuable insights for detecting CRP quality (Huang et al. 2025). Qin et al. (2024) combined FT‐NIR spectroscopy with a Long Short‐Term Memory (CNN‐LSTM) network model to classify CRP storage years, achieving 98.4% accuracy. Pu et al. (2023) utilized terahertz time‐domain spectroscopy combined with Convolutional Neural Network (CNN) models to distinguish CRP from different origins, where the fusion of spectra and image data (Add‐CNN) achieved the best performance. Chen, Li, et al. (2024) applied FT‐NIR spectroscopy with machine learning to distinguish CRP from adulterants (orange peel, mandarin rind), achieving over 99% classification accuracy and quantitative prediction of adulteration levels. Liu, Wang, et al. (2025) integrated hyperspectral imaging with CNN and PLS‐DA, combined with Competitive Adaptive Reweighted Sampling (CARS)‐based feature wavelength selection, to effectively identify CRP varieties and origins. Dai et al. (2023) used surface‐enhanced Raman spectroscopy (SERS) with PCA–LDA to classify CRP storage age, achieving 91% cross‐validated accuracy and noting a 1607 cm^*−*1^ peak inversely correlated with age. Tan et al. (2024) used portable NIR spectroscopy with PLS‐DA and wavelength selection to discriminate mold‐damaged CRP, Multiplicative Scatter Correction (MSC)‐PLS‐DA on outer‐surface spectra achieved 100% accuracy on both test and independent sets.

In recent years, many researchers have adopted Deep Neural Network (DNN) such as Wasserstein generative adversarial network (WGAN) and Residual Neural Network (ResNet) to combine with spectral datasets in food detection model, both to alleviate sample scarcity and to enhance the stability and robustness of classification models. Hu et al. (2024) used Wasserstein generative adversarial networks with gradient penalty (WGAN‐GP) for spectral and label augmentation in *kudzu* starch prediction, improving 1D‐CNN performance over PLSR and addressing sample limitations. Cui et al. (2024) combined Vis–NIR hyperspectral imaging with WGAN for data augmentation, improving CNN‐based prediction of soluble sugar content in cherry tomatoes. To address wheat moisture prediction Sun et al. (2025), employed Vis–NIR/SWIR hyperspectral imaging with WGAN, generating realistic spectra verified by t‐SNE and enhancing 1D‐CNN performance with feature selection methods such as Successive Projections Algorithm (SPA) and ReliefF. Yin et al. (2025) addressed limited‐sample challenges in fish maw authentication by applying Raman and NIR spectroscopy with WGAN, coupled with multi‐level fusion methods. The augmented data significantly enhanced 1D‐CNN classification, achieving 98.21% accuracy. Lyu et al. (2025) developed a conditional WGAN‐GP to synthesize hyperspectral reflectance for grape maturity and TSS. Augmenting with 2000 synthetic samples lifted 3D‐CNN test accuracy to 91%, and adding 250 samples improved 1D‐CNN regression performance. By applying FT‐NIR spectroscopy to *bolete* discrimination (Yan et al. 2022), reported that while PLS‐DA and Support Vector Machine (SVM) required spectral preprocessing to achieve high accuracy, ResNet directly handled raw data and delivered perfect classification performance. Zhang, Ai, et al. (2025) fused multispectral imaging with a fine‐tuned, pruned dual‐branch Convolutional‐LSTM‐ResNet, enabling early salt‐tolerance screening in Chinese cabbage with 95% validation accuracy and showcasing the gains of deep learning data fusion. Chen et al. (2022) compared PLS‐DA with data/feature‐level fusion to two‐dimensional correlation spectroscopy and ResNet for *bolete* identification, finding the deep model provides 100% accuracy without multiple preprocessing. Yu et al. (2025) demonstrated that 1D‐Inception‐ResNet can serve as a universal NIR model for multifruit quality assessment, improving robustness and accuracy over PLS/ELM (Extreme Learning Machine) while reducing reliance on handcrafted preprocessing.

These studies, which integrate spectroscopic with classification models, have demonstrated the potential of achieving rapid and non‐destructive detection in food authentication. However, different food matrices and detection requirements necessitate independent evaluation of suitable models, rather than simply adopting existing models. For CRP, it is essential to identify classification models that can accurately detect various types of counterfeiting. At present, most spectroscopic detection of CRP has focused on origin, storage age, and chemical composition, while research on the identification of multiple counterfeit types remains limited. Therefore, developing a rapid, non‐destructive, and precise model for detecting different methods of counterfeit CRP represents an urgent research priority.

**FIGURE 2:**
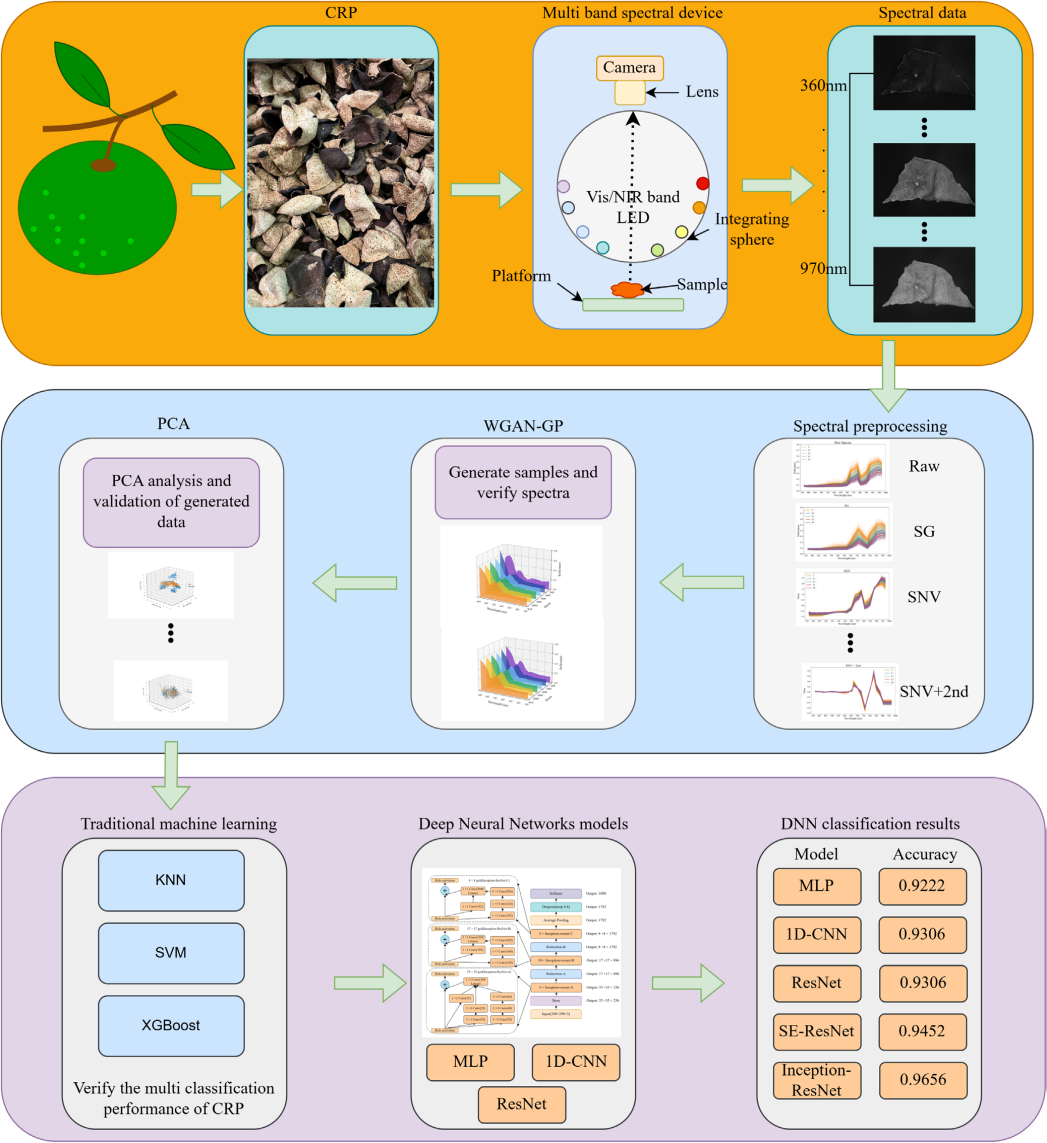
Workflow of this study and the proposed counterfeit CRP detection model.

To address this issue, the study proposes a non‐destructive detection model for counterfeit CRP by integrating Vis/NIR spectroscopy with deep learning algorithms. Spectral data of authentic CRP and four types of counterfeit CRP were collected using a Vis/NIR spectroscopy platform. The data were preprocessed to improve quality and representational capacity. WGAN‐GP was employed for data augmentation to enhance model generalization. The deep learning ResNet model was then applied to classify and detect the preprocessed data. The workflow of this study and the proposed model are illustrated in Figure 2. The main contributions of this study are as follows:

1. Authentic CRP and four types of counterfeit CRP were collected, and their Vis/NIR spectral data were acquired.
2. Spectral data were preprocessed, and key wavelength regions were analyzed. WGAN‐GP was used for sample augmentation to improve model stability, which was further validated by PCA and augmented spectral curves.
3. Deep learning models were analyzed to identify the most suitable model (Inception‐ResNet) for detecting different counterfeit CRP types, enabling efficient and precise non‐destructive authentication.

## 2 Materials and Methods

### 2.1 Preparation of Samples

In this study, two categories of CRP samples were collected: authentic CRP and counterfeit CRP. To broaden the applicability of the study, multiple types of counterfeit CRP were included. The authentic CRP samples were purchased from large pharmacies, with a total of 100 samples collected, denoted as T. The four types of counterfeit CRP were produced through different counterfeit methods: high‐temperature and high‐humidity fermentation, artificial dyeing, high‐temperature roasting, and acid‐alkali treatment. The four types of counterfeit CRP samples are denoted as F1, F2, F3, and F4, respectively. All four types of counterfeit CRP were designed to mimic the appearance, aroma, and color of authentic CRP. Although the counterfeit methods differed, each posed potential health risks. These counterfeit samples were obtained from CRP trading markets, with 200 samples in total (50 samples for each type). All samples originated from Xinhui, Guangdong, China, and were derived from the *Citrus reticulata* cultivar “Chachi.” Both authentic and counterfeit samples were analyzed and verified by experts in the field of food science to ensure their authenticity. To prevent cross‐contamination, all type samples were kept clean and individually stored in dry, transparent glass containers under cool indoor conditions at room temperature.

### 2.2 Spectral Data Acquisition

The Vis/NIR spectral acquisition system consisted of an integrating sphere, an industrial camera, a lens, a focusing drive unit, Vis/NIR Light Emitting Diode (LED) sources, a sample platform, and a computer. The Vis/NIR LEDs were positioned inside the integrating sphere, allowing reflected light to provide uniform illumination of the samples. The spectral range covered 360–970 nm, comprising 26 discrete wavelength bands. The industrial camera was equipped with an auto‐focusing lens, and the acquired images had a resolution of 1600 *×* 1200 pixels. The computer was used to control acquisition parameters and perform data analysis. The entire platform was integrated within a rigid outer frame to ensure stability.

Considering the characteristics of CRP, with both outer and inner peel surfaces, spectral data were collected from both sides of each sample, and the mean spectrum of the two surfaces was calculated for subsequent analysis. In total, 100 datasets were obtained from authentic CRP, while 200 datasets were collected from counterfeit CRP samples.

### 2.3 Data Preprocessing

#### 2.3.1 Black and White Correction

In this stage, before acquiring CRP spectral data, the black and white correction is used to eliminate errors introduced by reference deviations. The correction was performed using the following Equation (1):

(1) $$r=\frac{i-b}{w-b}$$

where *r* represents the corrected spectral image, *i* is the original spectral image, *b* denotes the spectral data measured from a black shield, and *w* corresponds to the spectral data measured from a white standard reference panel.

#### 2.3.2 Data Preprocessing

The spectral data were extracted and subjected to various preprocessing methods. The SG, first derivative, second derivative, and standard normal variate (SNV) were applied to preprocess the spectral data. These methods reduce the influence of random noise, baseline shifts caused by surface irregularities, and other unwanted interferences. In this study, multiple preprocessing methods were evaluated, including raw (unprocessed), SG, SNV, SG+1st derivative, SG+2nd derivative, SNV+1st derivative, and SNV+2nd derivative. Each preprocessing method was combined with subsequent classification models to analyze and identify the most suitable model for detecting counterfeit CRP.

#### 2.3.3 WGAN‐GP for Data Augmentation

To improve the generalization performance of classification models under limited sample conditions, WGAN‐GP was employed for data augmentation. In multi‐class counterfeit CRP classification, an imbalanced sample distribution can easily bias the model toward the majority classes. To mitigate this issue, WGAN‐GP was introduced to expand the dataset. This method incorporates the Wasserstein distance as the optimization objective and applies a gradient penalty mechanism, thereby enhancing both training stability and the diversity of generated samples. Compared with the standard GAN, WGAN‐GP is more suitable for limited sample scenarios, as it can generate synthetic CRP data that closely resembles the true distribution and better captures complex spectral features. Data augmentation using WGAN‐GP was conducted only on the training set. The generated synthetic samples were used exclusively to expand the training data, while the test set remained fully independent. By augmenting the dataset, the deep learning models achieved classification accuracy and robustness.

### 2.4 Classification Models

Among traditional machine learning algorithms, this study employed *K*‐nearest neighbor (k‐NN), SVM, and Extreme Gradient Boosting (XGBoost) as baseline classifiers. SVM has received considerable attention in CRP spectral analysis, as it maximizes the margin between classes and employs the Gaussian radial basis function (RBF) kernel. Although SVM is considered a traditional method, it has been widely applied. XGBoost, in comparison with conventional random forest algorithms, provides superior speed and robustness, making it particularly suitable for high‐dimensional spectral datasets. These models have already achieved promising results in food authentication tasks based on spectroscopy. Nevertheless, each model exhibits distinct characteristics and suitability for specific detection objectives.

Traditional machine learning models have demonstrated reliable performance in food quality detection based on spectroscopic data. However, their limitations remain evident when addressing the classification of multiple counterfeit CRP types. Although the counterfeit methods differ, the spectral curves of counterfeit CRP often exhibit substantial overlap, reflecting their fundamental chemical discrepancies with authentic CRP. To enhance the discriminative performance of Vis/NIR spectral data in counterfeit CRP detection, several deep learning models were used in this study.

**FIGURE 3:**
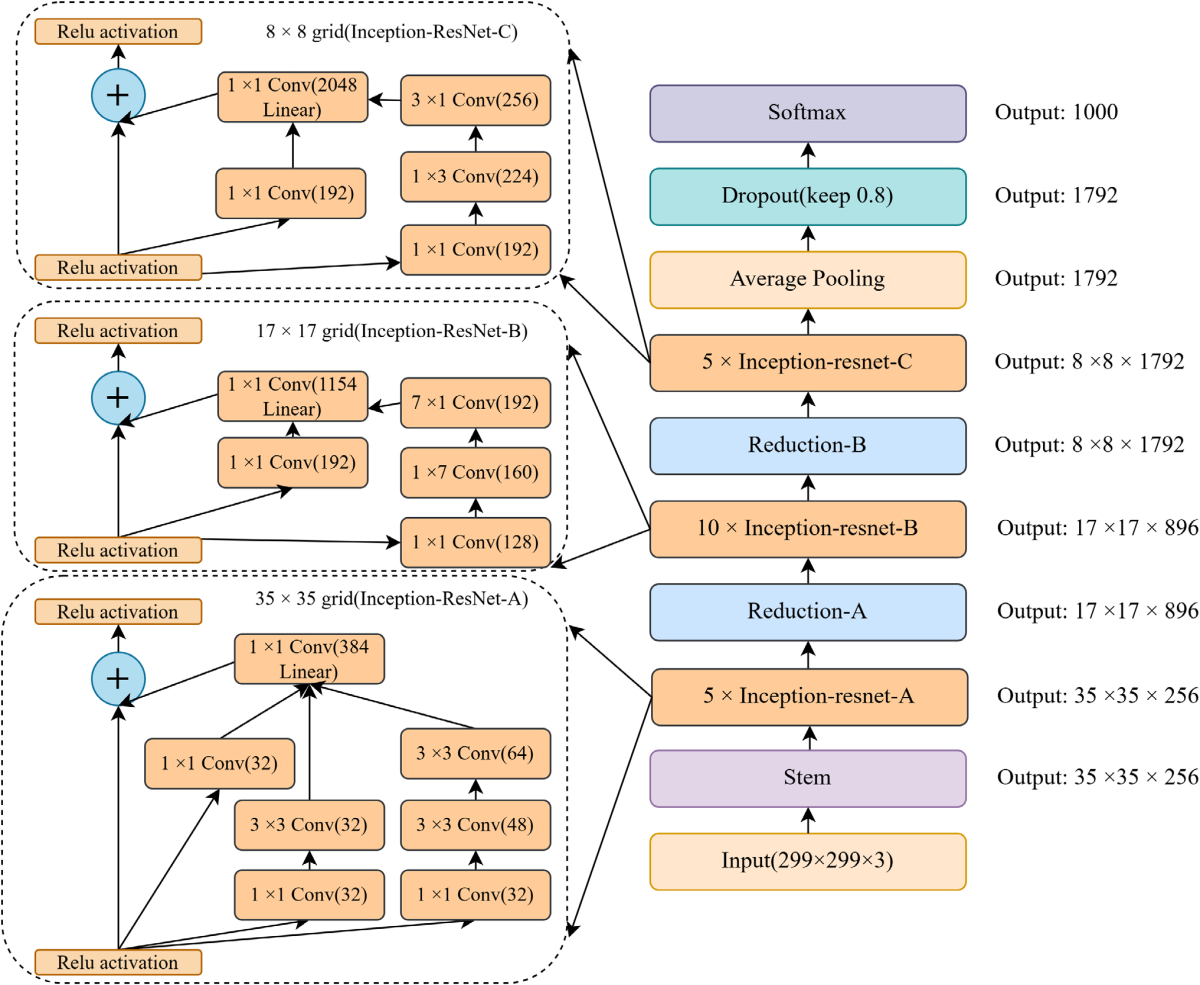
Architecture of the Inception‐ResNet network.

The one‐dimensional convolutional neural network (1D‐CNN) is particularly effective for sequential spectral data, as it captures local correlations and continuity across adjacent wavelengths, enabling the recognition of spectral variations induced by adulterated processing like chemical treatment or dyeing. The multilayer perceptron (MLP), a classical feed‐forward neural network, provides stable performance in nonlinear classification tasks and serves as a baseline to evaluate task complexity and feasibility. Residual Neural Networks (ResNet) address the vanishing gradient problem in deep architectures through residual connections, thereby facilitating the extraction of high‐level spectral features associated with different counterfeit types. The residual block can be expressed as Equation (2). The squeeze‐and‐excitation ResNet (SE‐ResNet) further incorporates a channel attention mechanism, adaptively enhancing responses to informative wavelengths while suppressing redundant or noisy features, thus improving sensitivity to subtle differences caused by high‐temperature processing or acid–alkali treatment (Kang et al. 2025). Finally, the Inception‐ResNet combines the multi‐scale feature extraction capability of the Inception module with the efficient gradient propagation of residual connections, achieving hierarchical representation of complex nonlinear features even within a limited spectral range. The Inception‐ResNet Network is illustrated in Figure 3. These models provide strengths in feature extraction and classification, providing comprehensive support for accurate identification of different counterfeit CRP types.

(2) $$y=F\left(x,\left\{W_{i}\right\}\right)+x$$

where *x* denotes the input feature, *F*(*x*, {*Wi*}) represents the residual mapping composed of several convolutional layers, normalization layers, and nonlinear activation functions, and *y* is the output obtained by residual addition.

To prevent potential data leakage, the original physical samples were first separated into training and test sets using a stratified splitting strategy. The test set was kept completely independent during the entire study and was not used for model training, parameter adjustment, or model selection.

### 2.5 Evaluation Metrics

Several commonly used metrics were employed to comprehensively evaluate the classification performance of the proposed models in counterfeit CRP detection. Accuracy reflects the overall correctness of predictions and provides an intuitive measure of the classifier's average performance across all samples. Precision indicates the proportion of correctly classified samples within those predicted as a given class, effectively reflecting the model's ability to control false positives. Recall emphasizes the proportion of correctly identified positive samples among all actual positives, making it suitable for assessing the risk of missed detections across different counterfeit types. The F1‐score, defined as the harmonic mean of precision and recall, balances the influence of false positives and false negatives, thus serving as a robust indicator of both stability and practical utility. The evaluation metrics are represented as Equations ((3), (4), (5), (6)). These metrics enable a multi‐dimensional evaluation of deep learning models for identifying various counterfeit CRP types under Vis/NIR spectroscopy.

(3) $$\text{Accuracy}=\frac{\mathrm{TP}+\mathrm{TN}}{\mathrm{TP}+\mathrm{TN}+\mathrm{FP}+\mathrm{FN}}$$

(4) $$\text{Precision}=\frac{\mathrm{TP}}{\mathrm{TP}+\mathrm{FP}}$$

(5) $$\;\text{Recall}=\frac{\mathrm{TP}}{\mathrm{TP}+\mathrm{FN}}$$

(6) $$\;F1-\text{score}=2\times \frac{\text{Precision}\times \text{Recall}}{\text{Precision}+\text{Recall}}$$

## 3 Results and Discussion

### 3.1 Chemical Composition and Spectral

**FIGURE 4:**
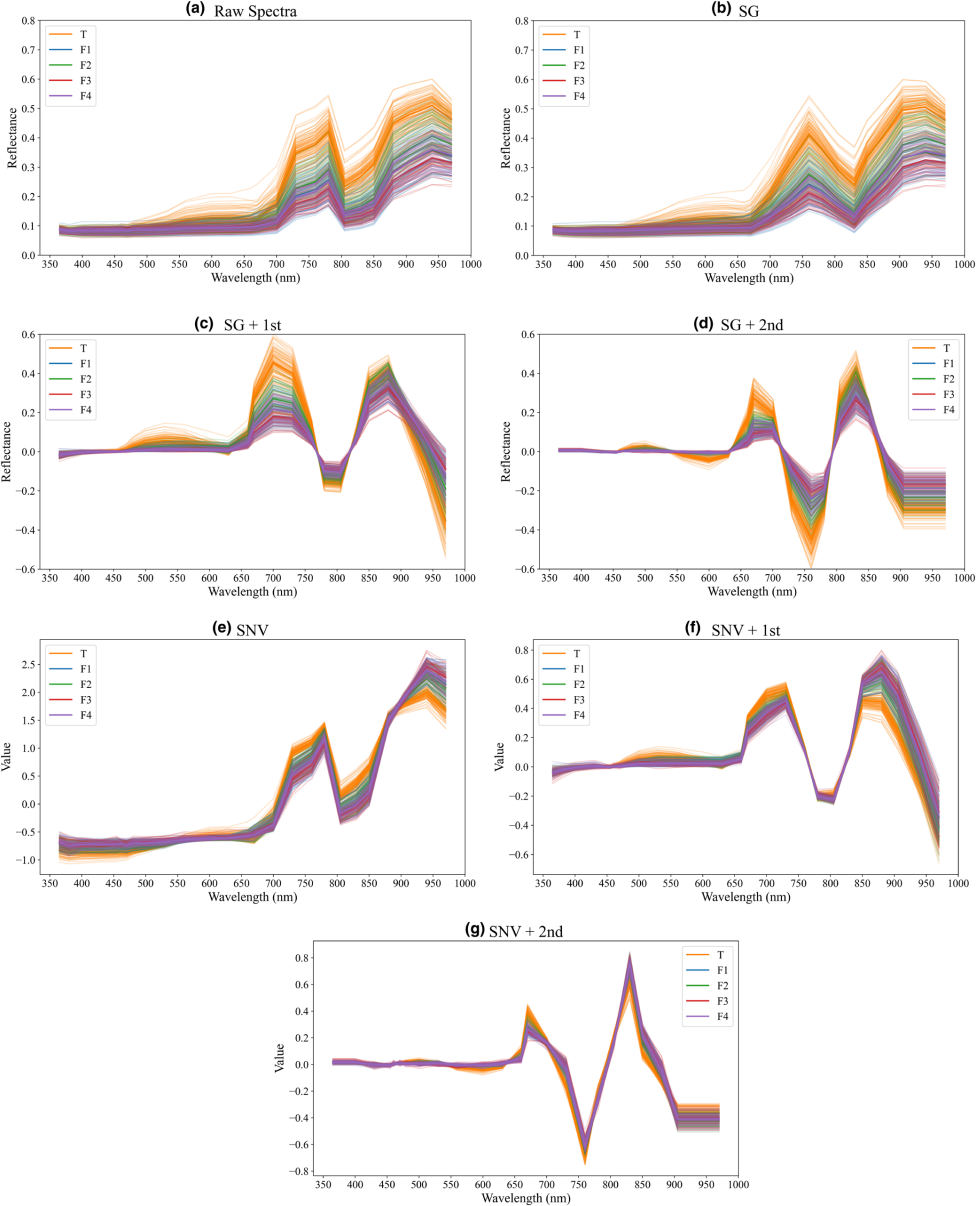
Raw and preprocessing comparison for Vis/NIR spectra: (a) Raw, (b) SG, (c) SG+1st, (d) SG+2nd, (e) SNV, (f) SNV+1st, (g) SNV+2nd. The authentic process CRP samples are denoted as T, the four types of counterfeit CRP samples are denoted as F1, F2, F3, and F4, respectively.

Vis/NIR spectroscopy shows advantages in reflecting the chemical composition of CRP, as its spectral features can be directly associated with major bioactive compounds. As shown in Figure 4, the authentic process CRP samples are denoted as T, whereas the four types of counterfeit CRP samples are denoted as F1, F2, F3, and F4, respectively. Flavonoids such as hesperidin, nobiletin, and polysaccharides may be associated with characteristic absorption bands in the 350–380 nm region, accompanied by scattering effects (Grassi et al. 2021). Chlorophyll residues showed a distinct Q‐band absorption signal around 600 nm, which may be associated with the weakening in samples subjected to high‐temperature fermentation or chemical treatment, highlighting the chlorophyll differences between authentic and counterfeit samples. In addition, the spectral region around 740–750 nm may be associated with O—H‐related absorptions and could reflect differences in polysaccharide‐related composition among samples. The spectral regions near 850 and 970 nm may reflect compositional differences among CRP samples and could be associated with C—H and O—H‐related absorptions (Ciurczak et al. 2021). These discriminative spectral regions were broadly consistent with compositional differences reported in previous studies and may provide a tentative chemical basis for distinguishing counterfeit CRP produced by different counterfeit methods.

### 3.2 Spectral Analysis

As shown in Figure 4, the raw spectra within the range of 360–970 nm exhibited typical reflectance features. Although differences across wavelengths were observed, the overall trends remained consistent, with considerable overlap among the four types of counterfeit CRP. After applying SG combined with first derivative processing (SG+1st), absorption peaks in the 700–900 nm region were significantly enhanced. In particular, authentic CRP samples (T) displayed a stronger peak around 750 nm, which was closely associated with differences in flavonoid and polysaccharide content. When the second derivative (SG+2nd) was further applied, spectral details became distinct, and subtle shifts in counterfeit samples near 850 and 970 nm were amplified.

SNV transformation effectively reduced scattering effects and aligned the spectral baselines of different CRP samples. In the SNV‐processed spectra, authentic CRP retained a signal in the 740–750 nm region corresponding to the third overtone of O–H vibrations, whereas counterfeit samples exhibited notably flattened curves, demonstrating differences in polysaccharide and water‐binding states. When combined with first or second derivative processing (SNV+1st, SNV+2nd), a distinct separation was observed between authentic and counterfeit CRP. Both SG and SNV preprocessing demonstrated strong effectiveness in highlighting compositional differences, making spectral discrepancies between authentic CRP and the four counterfeit types clearly distinguishable in the Vis/NIR region.

### 3.3 WGAN‐GP Augmentation Model Analysis

**FIGURE 5:**
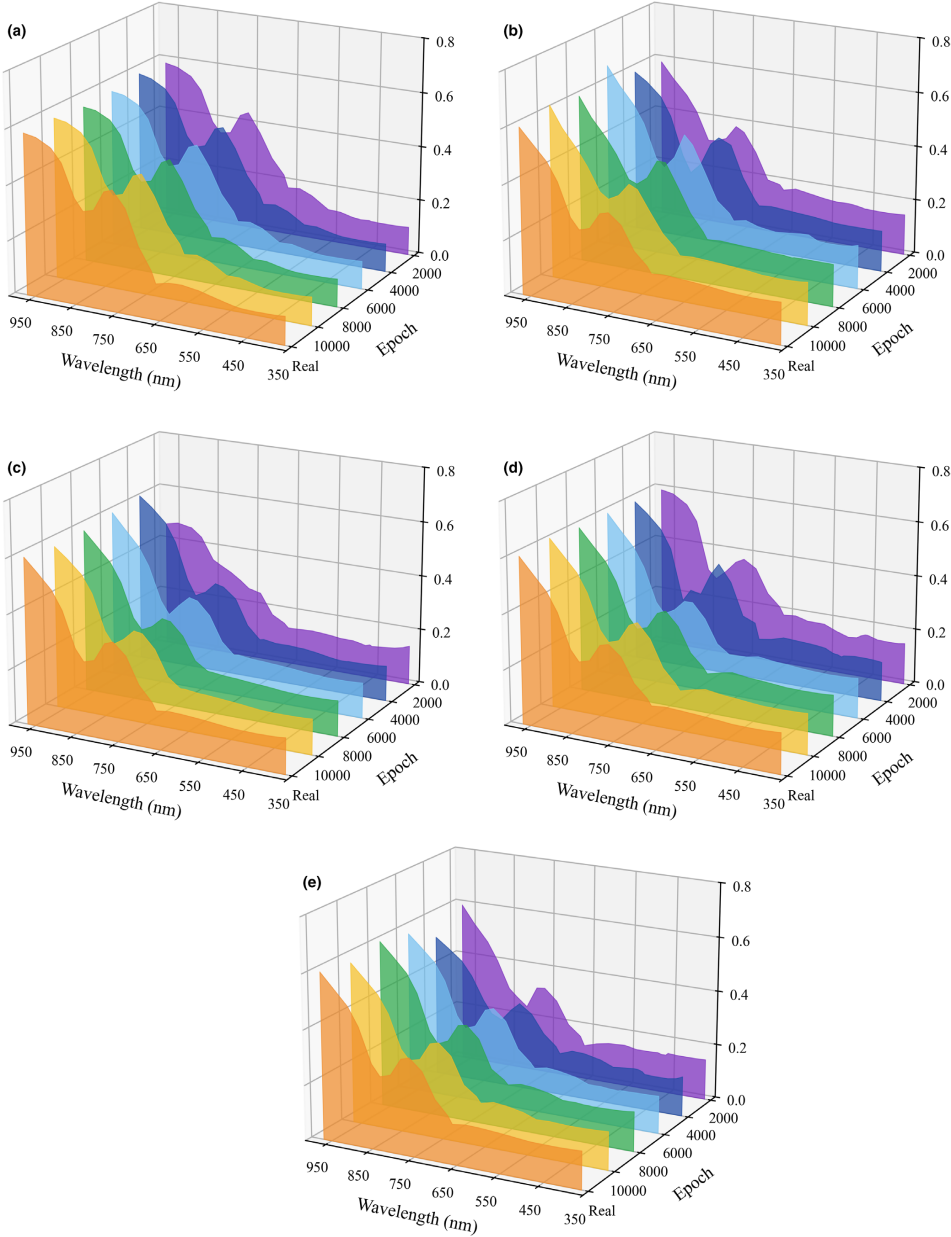
Comparison of spectral curves for five CRP samples (a–e: T, F1, F2, F3, F4): WGAN‐GP generated spectra at different epochs versus the corresponding real sample spectra.

**FIGURE 6:**
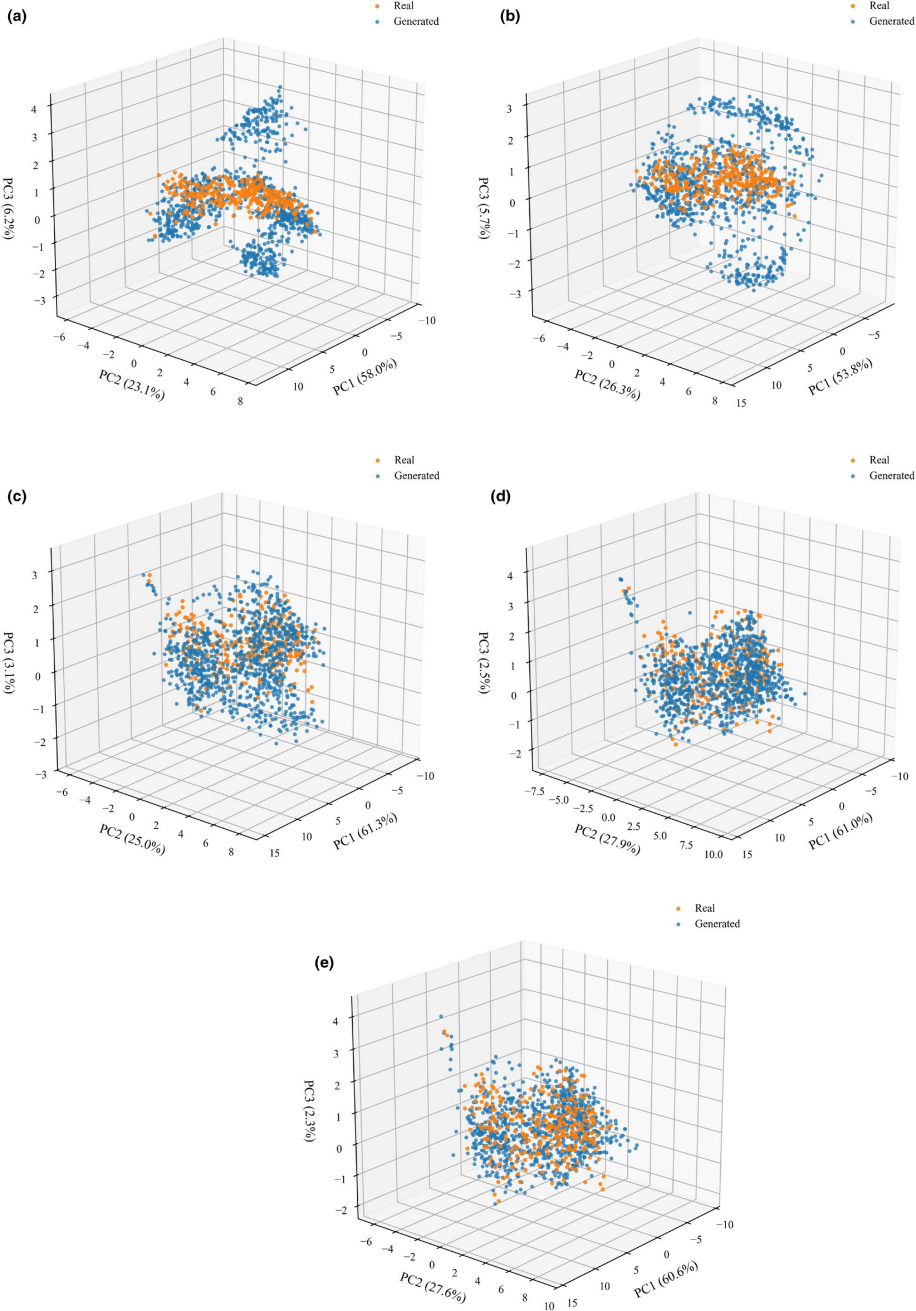
PCA three principal components comparison of WGAN‐GP generated with real CRP spectra for five samples (a–e: Epoch: 2000, 4000, 6000, 8000, 10,000).

The WGAN‐GP was applied to augment the limited CRP dataset, and its generative performance was validated using principal component analysis (PCA) and spectral curves. The original dataset contained 300 samples, which were expanded threefold to generate 900 additional samples, for a total of 1200 samples. The comparative spectral curves between WGAN‐GP‐generated data at different epochs and real samples are shown in Figure 5, and the corresponding PCA analysis is presented in Figure 6. The three‐dimensional PCA results showed that the real samples and generated samples overlapped extensively in the principal component space, with similar distribution points. This indicates that the generated samples effectively approximated the overall spectral of real CRP samples. As training iterations progressed from 2000 to 10,000 epochs, the aggregation between generated and real data increased, and the inter‐cluster gaps gradually diminished, reflecting the model's ability to progressively learn stable spectral feature representations during adversarial training.

To evaluate the fidelity of the WGAN‐GP generated spectra beyond PCA visualization, quantitative similarity metrics were calculated between real and generated CRP spectra, including mean spectrum MAE, mean spectrum RMSE, and MMD. As shown in Table 1, the generated spectra exhibited small reconstruction errors across all classes, with overall mean spectrum MAE and RMSE values of 0.015 and 0.023, respectively. The class‐wise MMD values also remained low, with an overall value of 0.033, indicating that the generated spectra reasonably preserved the distributional characteristics of the original data. These results provide support for the similarity between real and generated spectra.

**TABLE 1:** Quantitative similarity metrics between real and generated CRP spectra.

Class	Mean‐spectrum MAE	Mean‐spectrum RMSE	MMD
T	0.012	0.018	0.024
F1	0.015	0.022	0.031
F2	0.017	0.025	0.038
F3	0.014	0.021	0.029
F4	0.018	0.027	0.041
Overall	0.015	0.023	0.033

Further comparisons of average spectra across categories revealed that WGAN‐GP‐generated samples closely matched the real CRP samples in both peak positions and overall reflectance trends. Specifically, characteristic absorptions at 360 nm, around 600 nm, and within 740–970 nm were well reproduced. This consistency demonstrates that the generated data successfully represent the chemical compositional spectral curve differences among different counterfeit CRP types while avoiding mode collapse and distribution simplification. The introduction of WGAN‐GP not only alleviated overfitting risks associated with the limited datasets but also enhanced the generalization ability by providing augmented samples for discriminating the four types of counterfeit CRP.

### 3.4 Analysis of Classification Results

After spectral preprocessing, traditional machine learning models showed limited performance in classifying the four types of counterfeit CRP. As shown in Table 2, the results indicated that the accuracy, precision, recall, and F1‐scores of SVM, k‐NN, and XG‐Boost were mostly around 70%, falling limited of achieving precise discrimination. Differences were observed among categories; authentic CRP was classified relatively consistently, whereas the distinction between different counterfeit types often overlapped, restricting further improvement of model performance. Overall, traditional machine learning methods provided only basic discrimination capability in this study and were insufficient for the accurate identification of the four counterfeit CRP types.

**TABLE 2:** Classification results of traditional machine learning models.

Preprocess	Model	Train_accuracy	Train_precision	Train_recall	Train_fl	Test_accuracy	Test_precision	Test_recall	Test_fl
RAW	KNN	73.95%	73.95%	73.95%	73.95%	73.37%	73.82%	73.37%	73.59%
RAW	SVM	75.83%	75.83%	75.83%	75.83%	75.19%	75.42%	75.19%	75.30%
RAW	XGB	79.30%	79.30%	79.30%	79.30%	75.70%	75.90%	75.70%	75.80%
SG	KNN	73.18%	73.18%	73.18%	73.18%	73.37%	72.89%	73.43%	73.16%
SG	SVM	75.12%	75.62%	74.67%	74.92%	74.67%	75.13%	74.72%	74.92%
SG	XGB	79.08%	79.08%	79.08%	79.08%	75.44%	75.55%	75.44%	75.50%
SNV	KNN	76.68%	76.47%	76.68%	76.57%	75.19%	75.08%	74.90%	74.99%
SNV	SVM	73.44%	73.53%	73.44%	73.49%	73.11%	72.81%	73.11%	72.96%
SG+1st	KNN	75.25%	74.62%	75.25%	74.93%	75.19%	75.42%	75.06%	75.24%
SG+1st	SVM	74.67%	74.49%	74.67%	74.58%	74.15%	74.59%	74.15%	74.37%
SG+1st	XGB	77.52%	77.52%	77.52%	77.52%	76.48%	76.64%	76.48%	76.56%
SG+2nd	KNN	75.70%	75.31%	75.70%	75.50%	74.93%	74.97%	74.71%	74.84%
SG+2nd	SVM	74.47%	74.47%	74.47%	74.47%	74.41%	74.95%	74.64%	74.80%
SG+2nd	XGB	76.48%	76.90%	76.48%	76.69%	76.48%	76.64%	76.48%	76.56%
SNV+1st	KNN	77.52%	77.51%	77.52%	77.52%	75.96%	75.99%	75.96%	75.98%
SNV+1st	SVM	75.19%	75.17%	75.19%	75.18%	75.19%	75.74%	75.19%	75.46%
SNV+1st	XGB	76.81%	76.90%	76.81%	76.86%	76.74%	77.01%	76.74%	76.87%
SNV+2nd	KNN	77.52%	77.51%	77.52%	77.52%	75.70%	75.63%	75.70%	75.67%
SNV+2nd	SVM	75.12%	75.09%	75.12%	75.10%	75.19%	75.74%	75.19%	75.46%
SNV+2nd	XGB	77.78%	77.78%	77.78%	77.78%	76.48%	76.64%	76.48%	76.56%

**FIGURE 7:**
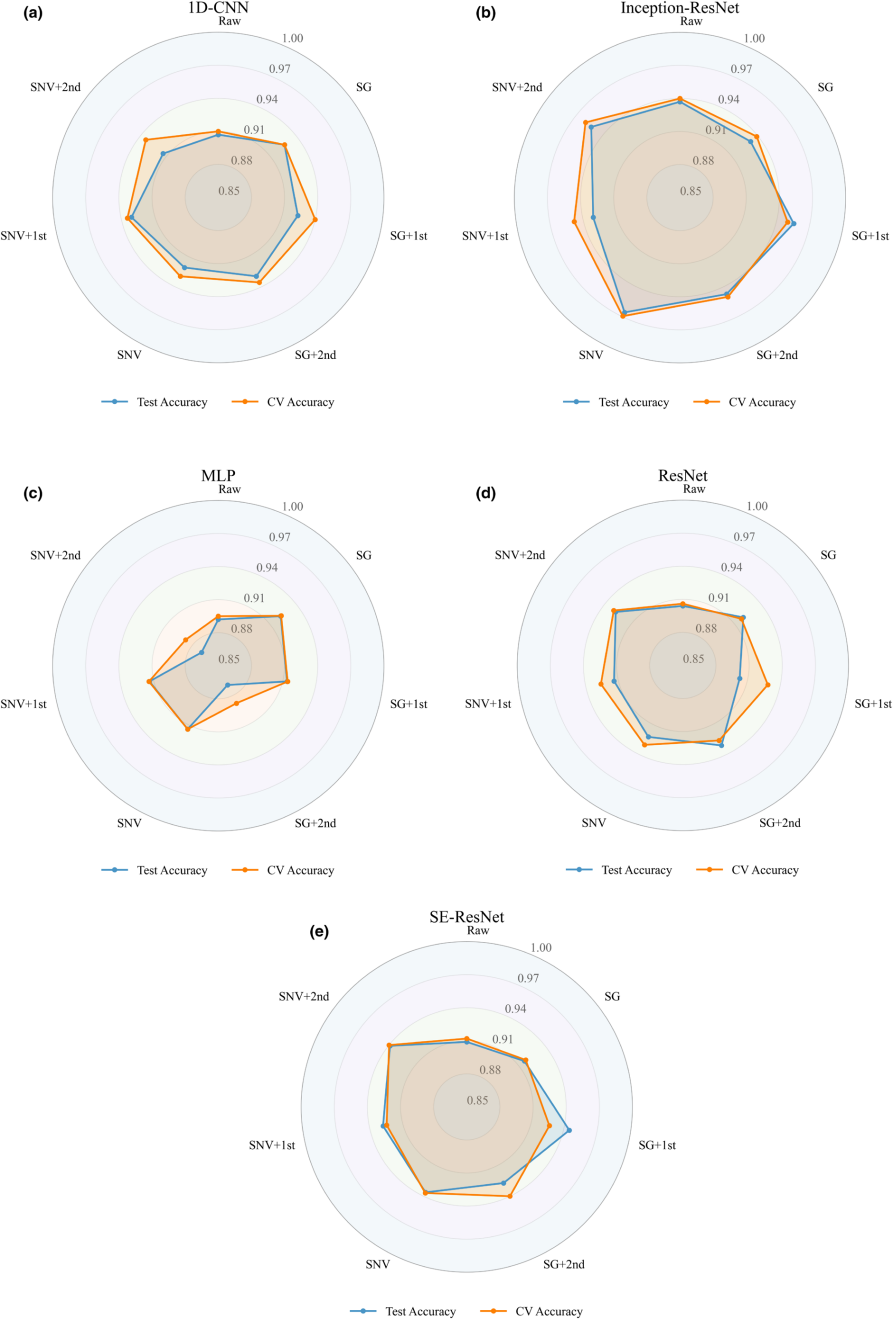
Deep learning classification results.

Under the same preprocessing conditions, the deep learning models achieved better results than the traditional machine learning algorithms. As shown in Table 3 and Figure 7, the results showed that Inception‐ResNet1D achieved the highest classification performance in most cases, with both test set and cross‐validation (CV) accuracies exceeding 96%. Precision, recall, and F1‐scores were also above 96%, demonstrating discriminative capability. ResNet, SE‐ResNet, and 1D‐CNN followed closely, each achieving average accuracies above 92%. These models also maintained relatively stable recall values, ensuring balanced recognition across different counterfeit types. While the overall accuracy of ResNet was slightly less than that of SE‐ResNet with attention mechanisms, it still clearly surpassed traditional machine learning models.

**TABLE 3:** Classification results of deep learning models.

Preprocess	Model	Train_acc	Train_prec	Train_rec	Train_f1	Test_acc	Test_prec	Test_rec	Test_f1
Raw	1D‐CNN	91.80% ± 0.42%	91.80% ± 0.55%	91.80% ± 0.48%	91.80% ± 0.51%	90.70% ± 0.72%	90.60% ± 0.96%	90.70% ± 0.84%	90.60% ± 0.88%
Raw	Inception‐ResNet	94.80% ± 0.34%	94.60% ± 0.43%	94.70% ± 0.38%	94.60% ± 0.40%	93.70% ± 0.58%	93.80% ± 0.76%	93.70% ± 0.66%	93.70% ± 0.70%
Raw	MLP	90.50% ± 0.52%	90.20% ± 0.66%	90.50% ± 0.59%	90.30% ± 0.62%	89.20% ± 0.84%	89.50% ± 1.08%	89.00% ± 0.96%	89.20% ± 1.01%
Raw	ResNet	91.50% ± 0.39%	91.60% ± 0.50%	91.40% ± 0.44%	91.50% ± 0.47%	90.40% ± 0.65%	90.50% ± 0.86%	90.20% ± 0.76%	90.40% ± 0.80%
Raw	SE‐ResNet	92.00% ± 0.35%	92.00% ± 0.45%	92.00% ± 0.39%	92.00% ± 0.42%	90.90% ± 0.61%	91.10% ± 0.81%	90.90% ± 0.71%	91.00% ± 0.76%
SG	1D‐CNN	92.94% ± 0.31%	92.94% ± 0.40%	92.94% ± 0.35%	92.94% ± 0.37%	92.67% ± 0.46%	92.41% ± 0.64%	92.50% ± 0.55%	92.45% ± 0.59%
SG	Inception‐ResNet	93.46% ± 0.30%	93.19% ± 0.39%	93.77% ± 0.34%	93.48% ± 0.36%	93.17% ± 0.44%	93.17% ± 0.61%	93.17% ± 0.53%	93.17% ± 0.57%
SG	MLP	92.15% ± 0.39%	91.83% ± 0.49%	92.15% ± 0.43%	91.99% ± 0.46%	92.22% ± 0.56%	92.92% ± 0.73%	92.67% ± 0.64%	92.68% ± 0.68%
SG	ResNet	92.22% ± 0.31%	92.48% ± 0.40%	92.10% ± 0.35%	92.29% ± 0.37%	92.00% ± 0.45%	92.00% ± 0.61%	92.00% ± 0.53%	92.00% ± 0.57%
SG	SE‐ResNet	91.57% ± 0.34%	91.77% ± 0.44%	91.72% ± 0.38%	91.75% ± 0.41%	91.67% ± 0.48%	91.67% ± 0.66%	91.67% ± 0.57%	91.67% ± 0.61%
SG+1st	1D‐CNN	94.85% ± 0.27%	94.52% ± 0.35%	94.28% ± 0.31%	94.40% ± 0.33%	92.39% ± 0.43%	92.39% ± 0.59%	92.39% ± 0.51%	92.39% ± 0.55%
SG+1st	Inception‐ResNet	97.38% ± 0.22%	97.38% ± 0.28%	97.38% ± 0.25%	97.38% ± 0.26%	95.56% ± 0.32%	95.56% ± 0.45%	95.56% ± 0.38%	95.56% ± 0.42%
SG+1st	MLP	92.36% ± 0.40%	92.36% ± 0.50%	92.36% ± 0.44%	92.36% ± 0.47%	91.39% ± 0.58%	91.39% ± 0.77%	91.39% ± 0.67%	91.39% ± 0.72%
SG+1st	ResNet	92.87% ± 0.38%	93.18% ± 0.49%	93.22% ± 0.43%	93.20% ± 0.46%	90.28% ± 0.71%	90.28% ± 0.97%	90.28% ± 0.85%	90.28% ± 0.91%
SG+1st	SE‐ResNet	94.71% ± 0.24%	94.71% ± 0.31%	94.71% ± 0.27%	94.71% ± 0.29%	94.52% ± 0.35%	94.52% ± 0.48%	94.52% ± 0.42%	94.52% ± 0.45%
SG+2nd	1D‐CNN	94.01% ± 0.28%	93.89% ± 0.36%	93.65% ± 0.32%	93.77% ± 0.34%	92.94% ± 0.39%	92.72% ± 0.54%	93.01% ± 0.47%	92.86% ± 0.50%
SG+2nd	Inception‐ResNet	97.50% ± 0.20%	97.50% ± 0.26%	97.50% ± 0.23%	97.50% ± 0.24%	94.72% ± 0.30%	94.72% ± 0.40%	94.72% ± 0.35%	94.72% ± 0.38%
SG+2nd	MLP	88.75% ± 0.50%	88.75% ± 0.64%	88.75% ± 0.57%	88.75% ± 0.60%	86.94% ± 0.91%	86.94% ± 1.17%	86.94% ± 1.03%	86.94% ± 1.09%
SG+2nd	ResNet	93.75% ± 0.24%	93.75% ± 0.31%	93.75% ± 0.27%	93.75% ± 0.29%	93.06% ± 0.30%	93.37% ± 0.41%	93.33% ± 0.36%	93.28% ± 0.38%
SG+2nd	SE‐ResNet	94.06% ± 0.29%	94.34% ± 0.37%	93.85% ± 0.33%	94.09% ± 0.35%	92.67% ± 0.41%	92.67% ± 0.56%	92.65% ± 0.49%	92.66% ± 0.52%
SNV	1D‐CNN	92.17% ± 0.31%	92.17% ± 0.40%	92.17% ± 0.35%	92.17% ± 0.37%	92.06% ± 0.43%	92.06% ± 0.58%	92.06% ± 0.51%	92.06% ± 0.54%
SNV	Inception‐ResNet	97.51% ± 0.18%	97.14% ± 0.24%	96.87% ± 0.21%	97.00% ± 0.22%	96.56% ± 0.24%	97.32% ± 0.33%	97.33% ± 0.29%	97.32% ± 0.31%
SNV	MLP	91.60% ± 0.39%	92.15% ± 0.49%	91.15% ± 0.43%	91.65% ± 0.46%	91.39% ± 0.57%	91.39% ± 0.76%	91.39% ± 0.66%	91.39% ± 0.71%
SNV	ResNet	92.43% ± 0.28%	92.43% ± 0.36%	92.43% ± 0.32%	92.43% ± 0.34%	92.20% ± 0.39%	92.40% ± 0.53%	91.95% ± 0.46%	92.17% ± 0.49%
SNV	SE‐ResNet	93.75% ± 0.24%	93.75% ± 0.31%	93.75% ± 0.27%	93.75% ± 0.29%	93.61% ± 0.32%	93.61% ± 0.44%	93.61% ± 0.38%	93.61% ± 0.41%
SNV+1st	1D‐CNN	93.75% ± 0.27%	93.75% ± 0.35%	93.75% ± 0.31%	93.75% ± 0.33%	93.06% ± 0.39%	93.06% ± 0.53%	93.06% ± 0.46%	93.06% ± 0.49%
SNV+1st	Inception‐ResNet	94.86% ± 0.28%	94.86% ± 0.36%	94.86% ± 0.32%	94.86% ± 0.34%	93.06% ± 0.43%	93.06% ± 0.59%	93.06% ± 0.51%	93.06% ± 0.55%
SNV+1st	MLP	92.36% ± 0.40%	92.36% ± 0.50%	92.36% ± 0.44%	92.36% ± 0.47%	91.39% ± 0.58%	91.39% ± 0.77%	91.39% ± 0.67%	91.39% ± 0.72%
SNV+1st	ResNet	93.19% ± 0.39%	93.19% ± 0.50%	93.19% ± 0.44%	93.19% ± 0.46%	93.19% ± 0.67%	91.59% ± 0.92%	91.38% ± 0.80%	91.49% ± 0.86%
SNV+1st	SE‐ResNet	93.06% ± 0.35%	93.06% ± 0.45%	93.06% ± 0.40%	93.06% ± 0.42%	92.78% ± 0.48%	92.50% ± 0.66%	92.88% ± 0.57%	92.69% ± 0.61%
SNV+2nd	1D‐CNN	91.25% ± 0.39%	91.25% ± 0.50%	91.25% ± 0.44%	91.25% ± 0.46%	91.39% ± 0.56%	91.39% ± 0.76%	91.39% ± 0.66%	91.39% ± 0.71%
SNV+2nd	Inception‐ResNet	97.64% ± 0.24%	97.64% ± 0.31%	97.64% ± 0.27%	97.64% ± 0.29%	95.28% ± 0.34%	95.28% ± 0.46%	95.28% ± 0.40%	95.28% ± 0.43%
SNV+2nd	MLP	88.75% ± 0.50%	88.75% ± 0.64%	88.75% ± 0.57%	88.75% ± 0.60%	86.94% ± 0.91%	86.94% ± 1.17%	86.94% ± 1.03%	86.94% ± 1.09%
SNV+2nd	ResNet	94.58% ± 0.31%	94.58% ± 0.40%	94.58% ± 0.35%	94.58% ± 0.37%	92.78% ± 0.47%	92.78% ± 0.63%	92.78% ± 0.55%	92.78% ± 0.59%
SNV+2nd	SE‐ResNet	93.68% ± 0.29%	93.33% ± 0.37%	93.00% ± 0.33%	93.16% ± 0.35%	93.89% ± 0.40%	94.11% ± 0.54%	94.00% ± 0.47%	94.01% ± 0.50%

**FIGURE 8:**
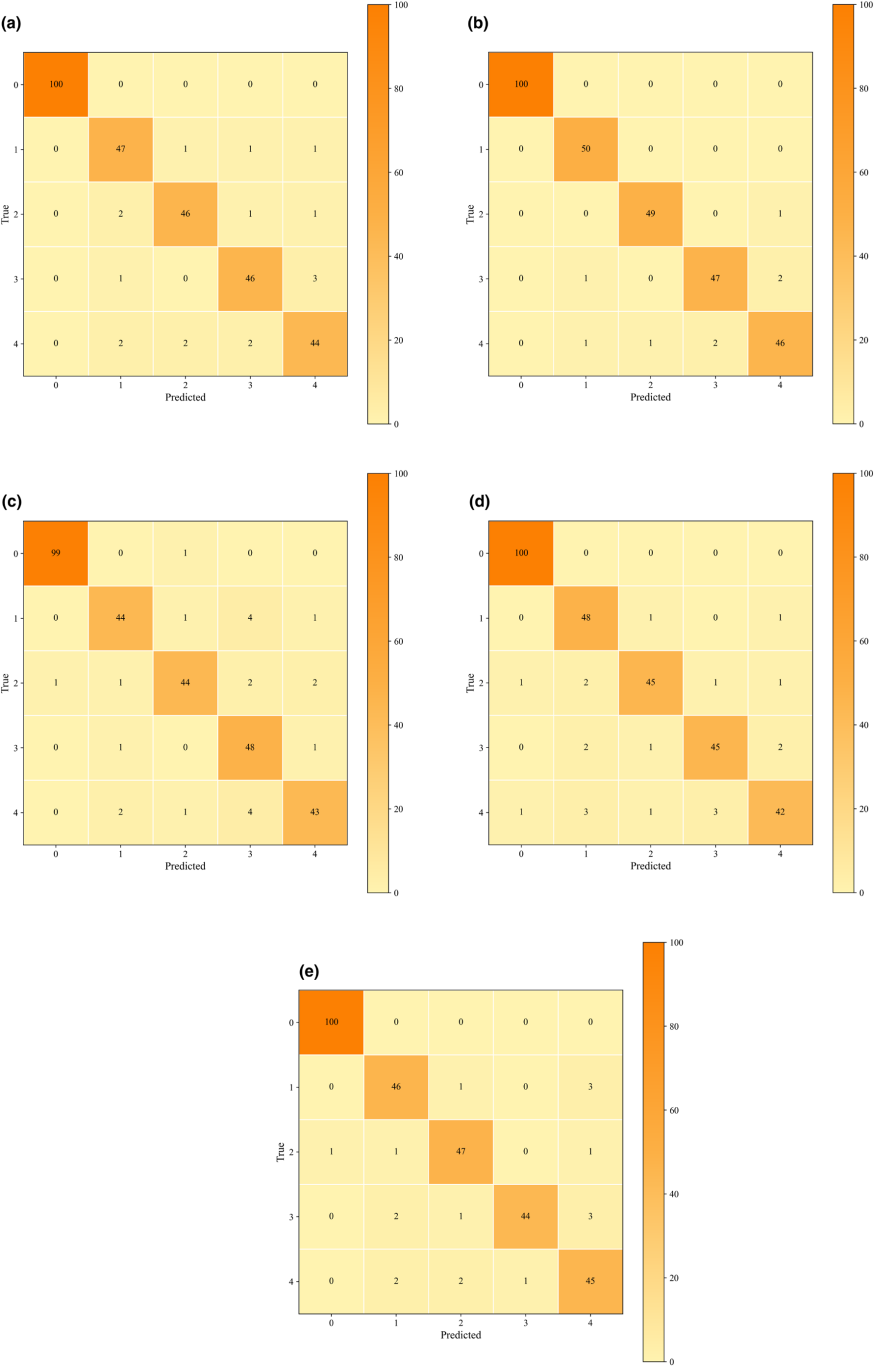
Confusion matrices of deep learning models, (a) 1D‐CNN, (b) Inception–ResNet1D, (c) MLP, (d) ResNet1D, and (e) SE–ResNet1D.

In comparison, MLP exhibited relatively limited performance, with accuracies typically ranging between 88% and 91%, indicating a weaker ability to capture complex spectral features compared with other deep neural network architectures. Overall, deep learning models delivered significant improvements across accuracy, precision, recall, and F1 metrics, enabling high‐precision discrimination of the four counterfeit CRP types. Figure 8 illustrates the confusion matrices of deep learning models for the classification of multiple counterfeit types of CRP.

To examine the practical contribution of WGAN‐GP augmentation, an ablation experiment was conducted under the best preprocessing setting for each deep learning model. As shown in Table 4, all evaluated models achieved higher accuracy after introducing WGAN‐GP generated samples. The accuracy improvements ranged from 1.39% to 2.39%, with the largest gain observed for Inception‐ResNet under SNV preprocessing. These results indicate that WGAN‐GP augmentation provided useful Supporting Information under the limited sample setting and contributed to improved classification performance.

**TABLE 4:** Ablation experiment of WGAN‐GP under the best preprocessing setting for each deep learning model.

Model	Best preprocessing	Without WGAN‐GP accuracy (%)	With WGAN‐GP accuracy (%)
1D‐CNN	SNV+1st	91.53 ± 0.72	93.06 ± 0.55
Inception‐ResNet	SNV	94.17 ± 0.64	96.56 ± 0.43
MLP	SG	90.83 ± 0.81	92.22 ± 0.67
ResNet	SNV+1st	91.67 ± 0.77	93.19 ± 0.58
SE‐ResNet	SG+1st	92.78 ± 0.69	94.52 ± 0.49

An additional external validation experiment was carried out using newly collected CRP samples from different regions, cultivars, and batches. This external dataset also included one authentic CRP class and four counterfeit CRP classes produced by different counterfeit methods, with 25 samples collected for each class, giving a total of 125 samples. All of these samples were fully separated from the original training and test sets. Their data were acquired under the same environmental conditions and with the same hardware and software settings. The trained models were subsequently evaluated independently on this external dataset.

As shown in Table 5, the external validation results demonstrated that the proposed method maintained effective classification performance. Across different models, the best external validation accuracies ranged from 80.00% to 93.00%. Among all evaluated settings, Inception‐ResNet under SNV preprocessing achieved the highest external validation performance, with an accuracy of 93.00%, a precision of 93.10%, a recall of 93.00%, and an F1‐score of 92.85%. Notably, this result remained consistent with the optimal model identified in the original experiment, further supporting the stability and reliability of the model selection conclusion. These results indicate that the proposed method retained promising effectiveness when applied to samples collected from different regions, cultivars, and batches.

**TABLE 5:** Best external validation results of different deep learning models.

Model	Preprocessing	Test accuracy (%)	Test precision (%)	Test recall (%)	Test F1 (%)
1D‐CNN	SNV	80.00% ± 1.37%	80.24% ± 1.11%	80.00% ± 1.42%	78.33% ± 1.28%
Inception‐ResNet	SNV	93.00% ± 0.68%	93.10% ± 0.74%	93.00% ± 0.61%	92.85% ± 0.79%
ResNet	SNV	89.00% ± 0.92%	89.83% ± 0.86%	86.00% ± 1.27%	84.69% ± 1.18%
SE‐ResNet	SG+1st	88.00% ± 1.05%	87.78% ± 0.94%	88.00% ± 1.12%	85.04% ± 1.21%
MLP	SG	84.00% ± 1.43%	87.60% ± 1.08%	84.00% ± 1.36%	84.67% ± 1.17%

False Classification Rate (FCR) was additionally introduced as an error centric metric in this study (Rana et al. 2026). Under the optimal internal test condition of SNV—Inception‐ResNet, the model achieved an FCR of 3.44%. In the WGAN‐GP ablation experiment, FCR decreased from 5.83% without augmentation to 3.44% with augmentation. In the external validation experiment, the best setting still achieved an FCR of 7.00%. These results further support the practical reliability of the proposed model.

### 3.5 Discussion

The experimental results demonstrated that spectral preprocessing is efficient in enhancing the performance of classification models. Compared with the raw spectra, the application of SG or combined with first or second derivative processing strengthened spectral features and improved model discrimination in key regions such as 740–970 nm. SNV effectively reduced scattering effects and aligned spectral baselines across samples, thereby improving feature comparability. Among the preprocessing methods evaluated, SNV exhibited the most favorable overall performance.

In comparison with SG and its derivatives, SNV was more effective in eliminating variability arising from differences in particle size, CRP surface roughness, and scattering intensity, which substantially enhanced feature consistency. Although SG and its first and second derivative variants amplified spectral details, they also introduced additional noise, which interfered with classification performance in certain wavelength regions. By contrast, SNV not only suppressed scattering effects but also reduced inter‐class overlap while preserving the main absorption patterns, thus achieving higher classification accuracy than other methods. Further analysis revealed that SNV combined with first or second derivative processing (SNV+1st, SNV+2nd) sometimes produced excessive oscillations in specific regions, which weakened the discrimination between categories. Overall, SNV proved particularly suitable for CRP spectra, where physical sample characteristics strongly influence measurement quality, and it was identified as the key preprocessing method to ensure improved model performance.

WGAN‐GP was employed to mitigate the limitations caused by the limited number of samples. The generative results indicated that this method could partially compensate for the scarcity of real data. PCA analysis showed that the distribution of generated spectra gradually converged with that of the real spectra, demonstrating that the model successfully learned the underlying distribution characteristics of CRP spectra during training. At the same time, the generated spectra maintained consistent absorption patterns with the real samples at key wavelengths, indirectly confirming the effectiveness of WGAN‐GP in preserving essential chemical component information. These results demonstrate the practical value of WGAN‐GP in expanding CRP spectral datasets. However, the generated data should be regarded as a supplement to, rather than a substitute for, authentic samples.

By integrating the experimental results from different preprocessing methods and models, it was demonstrated that Inception‐ResNet achieved the most superior performance across multiple evaluation metrics. The accuracy on the test set consistently remained above 93%, and for most preprocessing combinations exceeded 95%, reaching up to approximately 96%. Precision, recall, and F1‐scores also remained highly consistent, reflecting the robustness and reliability of this model in multi‐class counterfeit CRP identification. Compared with other deep learning models, Inception‐ResNet demonstrated discriminative ability by fully extracting multi‐scale spectral features and maintaining stable training through residual connections. This indicates that Inception‐ResNet provides the best performance in authenticating CRP using Vis/NIR spectroscopy combined with deep learning.

The other deep learning models also demonstrated advantages in this study. The 1D‐CNN achieved classification accuracies above 92% under most preprocessing conditions, benefiting from its simple structure and strong adaptability in extracting local spectral patterns. SE‐ResNet, by introducing a channel attention mechanism, generally reached 92%–94% in accuracy, precision, recall, and F1‐scores. Compared with standard ResNet, SE‐ResNet more effectively emphasized informative wavelengths, thereby improving sensitivity in the identification of counterfeit CRP samples. The performance of ResNet was slightly lower, with accuracy maintained at around 90%. In contrast, the MLP exhibited relatively limited capability, with accuracy ranging mainly from 86% to 92%. This reflects its limited ability to capture complex spectral features. Although the performance varied across different models, all deep learning algorithms substantially outperformed traditional machine learning methods. These results confirm the suitability of deep neural networks for CRP spectral data modeling and multi‐class counterfeit detection.

Compared with deep learning methods, traditional machine learning models demonstrated limitations in this study. Although traditional algorithms have advantages in low‐dimensional or linearly separable cases, their capacity for feature extraction and discrimination was insufficient when addressing the complex multi‐class counterfeit CRP problem. These models failed to capture subtle critical compositional differences in the spectra. Therefore, traditional methods are more suitable as baseline algorithms, but they fall short of meeting the practical requirements for high accuracy and robustness in counterfeit CRP detection.

It should be noted that the present study did not include direct compositional quantification or feature importance analysis. Therefore, the spectral interpretations provided here should be regarded as tentative and literature supported rather than definitive chemical evidence. Future work should incorporate direct compositional measurements and model‐based feature importance analysis to further strengthen the chemical interpretability of the proposed method.

From a practical perspective, the Vis/NIR model also differs from previously reported FT‐NIR approaches in both sample handling and deployment. Many FT‐NIR studies are conducted on powdered materials or other small and highly controllable sample forms, which facilitate spectral acquisition but do not preserve sample integrity. In contrast, CRP is commonly processed, traded, stored, sold, and used in relatively intact piece form. Under such conditions, the Vis/NIR framework used in this study is more suitable for direct intact sample acquisition. In addition, compared with FT‐NIR systems, the Vis/NIR platform used here has a lower equipment cost, which may further improve its practicality in cost‐sensitive settings. Therefore, even though previous FT‐NIR studies have achieved very high accuracies, evaluation under Vis/NIR conditions remains meaningful. This model better matches the practical need for non‐destructive detection of CRP samples.

In summary, SNV proved to be the most effective preprocessing method. Deep learning models demonstrate superiority over traditional machine learning algorithms. Inception‐ResNet achieved the best performance across accuracy, precision, recall, and F1‐score. The introduction of WGAN‐GP partially alleviated the limitations of sample size, and the close agreement between generated and real spectra at key wavelengths validated its effectiveness.

## 4 Conclusion

Counterfeiting of CRP is widespread and continues to evolve, leading to severe economic losses and health risks. In this study, Vis/NIR spectroscopy combined with deep learning algorithms was applied to the identification and classification of four types of counterfeit CRP. The results demonstrated that SNV was the most effective preprocessing method, providing stable input for subsequent feature extraction. The WGAN‐GP effectively alleviated the problem of limited sample size and improved the generalization ability of the models. Among them, the deep learning model Inception‐ResNet achieved the best performance across all evaluation metrics, exhibiting both high classification accuracy and robustness.

This study proposes an efficient, non‐destructive, and accurate model for counterfeit CRP detection. The study not only provides theoretical and technical support for CRP quality control and market supervision but also offers new insights into the broader application of spectroscopy in food authentication and quality evaluation. Future work will focus on developing evaluation metrics for spectral data generation and validating a more efficient model. In addition, incorporating prior chemical knowledge and multimodal information will further enhance model robustness and interpretability in addressing increasingly complex counterfeiting methods.

## Author Contributions

**Sudan Chen:** investigation, validation, data curation. **Chao Ma:** conceptualization, supervision, project administration, resources, writing – review and editing, validation. **Zhiyong Dai:** conceptualization, visualization, software. **Mingkun Zhang:** writing – original draft, methodology, visualization, formal analysis, data curation, software. **Mingtong Du:** software, formal analysis, resources. **Jianwei Ma:** funding acquisition, project administration, supervision, visualization. **Yunxia Yuan:** investigation, validation, methodology.

## Funding

This work was supported by Aeronautical Science Foundation of China (20200051042003), the Key Science and Technology Research Project of Henan Province (232103810040), and the Key Scientific Research Project of Higher Education Institutions in Henan Province (24A550007).

## Conflicts of Interest

The authors declare no conflicts of interest.

## Data Availability

The data that support the findings of this study are available from the corresponding author upon reasonable request.
